# The Construction of Heterothallic Strains of *Komagataella kurtzmanii* Using the I-SceI Meganuclease

**DOI:** 10.3390/biom15010097

**Published:** 2025-01-10

**Authors:** Daria D. Sokolova, Philipp I. Akentyev, Kristina O. Petrova, Lyudmila V. Lyutova, Aleksei A. Korzhenkov, Irek I. Gubaidullin, Stepan V. Toshchakov, Dmitry G. Kozlov

**Affiliations:** National Research Center “Kurchatov Institute”, 123182 Moscow, Russia

**Keywords:** *Komagataella kurtzmanii*, *Komagataella phaffii*, heterothallic yeast, cell mating type, I-SceI meganuclease, repair, genome editing

## Abstract

The methylotrophic yeast *Komagataella kurtzmanii* belongs to the group of homothallic fungi that are able to spontaneously change their mating type by inversion of chromosomal DNA in the MAT locus region. As a result, natural and genetically engineered cultures of these yeasts typically contain a mixture of sexually dimorphic cells that are prone to self-diploidisation and spore formation accompanied by genetic rearrangements. These characteristics pose a significant challenge to the development of genetically stable producers for industrial use. In the present study, we constructed heterothallic strains of *K. kurtzmanii*, ensuring a constant mating type by unifying the genetic sequences in the active and silent MAT loci. To obtain such strains, we performed site-directed inactivation of one of the two yeast MAT loci, replacing its sequence with a selective HIS4 gene surrounded by I-SceI meganuclease recognition sites. We then used transient expression of the SCE1 gene, encoding a recombinant I-SceI meganuclease, to induce site-specific cleavage of HIS4, followed by damage repair by homologous recombination in mutant cells. As a result, heterothallic strains designated ‘Y-727-2(alpha)’ and ‘Y-727-9(a)’, which correspond to the α and **a** mating type, respectively, were obtained. The strains demonstrated a loss of the ability to self-diploidize. The results of PCR and whole genome analysis confirmed the identity of the contents of the MAT loci. Analysis of the genomes of the final strains, however, revealed a fusion of chromosome 3 and chromosome 4 in strain Y-727-2(alpha)-1. This finding was subsequently confirmed by pulsed-field gel electrophoresis of yeast chromosomes. However, the ability of the Y-727-2(alpha)-derived producers to efficiently secrete recombinant β-galactosidase was unaffected by this genomic rearrangement.

## 1. Introduction

The classical variant of yeast hybridization involves the interaction of haploid cells of opposite mating types. The process is mediated by secreted peptide pheromones and their receptors. The genes encoding regulatory proteins that determine the type of cell mating are located in the *MAT* loci [1,2].

In yeasts with secondary homothallism, the *MAT* genes are represented by alternative alleles *MAT**a*** and *MATα*, encoding genes of a and α mating types, respectively. While one of the alleles is active, the genes of the other are located near the heterochromatic region and are subject to repression. Replacement of an allele at the active locus results in a switch of the sexual phenotype. The mechanisms of this switch, whether spontaneous or dependent on certain conditions, differ between species of homothallic yeast [1,3].

In *S. cerevisiae*, the alteration of mating type is initiated by the HO endonuclease, which performs double-strand DNA breaks at the active *MAT* locus, which contains the *MAT**a*** or *MATα* genes [4]. The *HMLα* and *HMR**a*** alleles, which represent the *MATα* and *MAT**a*** alleles, respectively, are located at opposite termini of chromosome III in an inactive, repressed state within the heterochromatic chromosome region [1]. The hydrolysis of chromosomal DNA in the region of the active *MAT* locus induces repair by homologous recombination, utilizing either the *HMLα* or *HMR**a*** locus sequences as matrices. This event has a 50% probability of reversing the mating type of the cell. Laboratory strains of *S. cerevisiae* are typically heterothallic, and contain mutations in the *HO* gene or carry its recessive allele *ho* [5].

The mating-type switch in *K. phaffii* is subject to regulation by an alternative mechanism that is active on glucose-containing media [6]. The mechanism is based on the inversion of chromosomal DNA, which leads to a switch in the localization of the MATα and MATa alleles, placing one of them in the telomeric region of the chromosome and repressing its transcription through the telomere position effect. It is hypothesized that inversions represent an ancestral mode of cell sex switching, preceding the mechanism observed in the yeast *S. cerevisiae* [3]. In the yeast *K. phaffii*, both *MATα* and *MAT**a*** alleles are located on chromosome IV, surrounded by *SLA2* and *DIC1* genes as part of inverted repeats involved in recombination, resulting in chromosomal DNA inversion [3]. The structure of the *MAT* loci, as well as the functional activity of the *MAT**a1***, *MAT**a2***, *MATα1* and *MATα2* genes, and their role in the mating and sporulation processes of the yeast *K. phaffii*, are reviewed in detail by Heistinger et al. [7]. In particular, the main function of the *MAT**a2*** and *MATα1* genes is realized at the stage of diploid cell formation, while the *MAT**a1*** and *MATα2* genes control the sporulation process [7]. The *DIC1* gene separates the silent *MAT* locus from the telomere, both of which are inhibited by the telomeric chromatin structure [8]. The active and silent mating-type (*MAT*) alleles are separated by a DNA fragment of approximately 135,000 base pairs containing the centromere. It has been demonstrated that in *K. phaffii* cells, recombination, which results in chromosomal inversion and mating-type switch, occurs within the *DIC1* gene region [3,6]. Deletions in the *DIC1* region has been demonstrated to impede chromosomal inversions, leading to the generation of heterothallic *K. phaffii* strains [7].

The meganuclease I-SceI is a homing endonuclease and is responsible for intron mobility in *S. cerevisiae* mitochondria [9,10]. The I-SceI recognition site is constituted by an 18 bp sequence (TAGGGATAACAGGGGTAAT) [11], which accounts for the rare occurrence of the site in genomic DNA. This property, in conjunction with the high functional activity of this nuclease, has resulted in its extensive application for genetic manipulations of both microbes and more complex eukaryotic organisms [12,13,14]. For example, it has been used in *S. cerevisiae* cells to achieve directed and highly efficient integration of recombinant DNA [15]. It was also used to perform multiple sequential integration and deletion of a marker gene cassettes flanked by I-SceI recognition sites [16]. I-SceI directed hydrolysis of chromosomal DNA was used to delete the *pyrG* marker and stimulate directed integration of recombinant DNA in *Trichoderma reesei* cells [17]. Meganuclease has been successfully used for genetic manipulation of *Streptomyces coelicolor*, *Pyricularia oryzae*, *Corynebacterium glutamicum* and other microorganisms [18,19,20]. However, to our knowledge, the use of I-SceI in *K. phaffii* or *K. kurtzmanii* cells has not been reported. At the same time, the genomes of these yeasts do not contain natural I-SceI recognition sites, which is a necessary prerequisite and makes genetic manipulations with these microorganisms using this meganuclease both convenient and reliable.

A gene expression system with high efficiency has been developed based on the methylotrophic yeast *K. kurtzmanii* as an alternative to *K. phaffii* [21,22,23]. The yeast species *K. kurtzmanii* and *K. phaffii* are the most closely related within the genus *Komagataella*, exhibiting a level of genomic homology of 98% [7,24]. Thus, it can be reasonably inferred that mating-type change and hybridization in *K. kurtzmanii* and *K. phaffii* are carried out in a similar manner. Consequently, as a result of the homothallic status, natural strains of *K. kurtzmanii* and their laboratory derivatives contain a mixture of different-sexed cells, which are capable of uncontrolled crossing/sporulation and, therefore, genetic changes that jeopardize the prospects of their stable industrial use.

In this regard, our primary objective was to obtain heterothallic *K. kurtzmanii* strains that were devoid either of the ability to uncontrollably change the mating type, as occurs in wild-type strains, or the possibility to lose its mating identity, as occurs in strains mutant for the *MAT* loci. To obtain the desired strains, we chose to pursue an approach in which the constancy of cell mating type would be ensured by unifying the contents of the *MAT* loci. This approach represents an alternative to the previously described method, which was based on the inactivation of the chromosomal inversion mechanism [25].

## 2. Materials and Methods

### 2.1. Plasmid Construction

Escherichia coli strain Top10 (Invitrogen, Carlsbad, CA, USA) was used for cloning and amplification of plasmids. *E. coli* transformants were obtained on dishes with agarised LB medium, consisting of 25 g/L LB Broth (VWR International, Darmstadt, Germany), 2% agar (E-406, Russia) and ampicillin (Synthesis, Russia) with a final concentration of 100 µg/mL. For the isolation of plasmid DNA, the transformed cells were cultured on a rotary shaker at 37 °C for 18 h at 250 rpm on LB medium supplemented with ampicillin to a concentration of 100 μg/mL. Plasmid DNA was isolated using the Cleanup S-Cap plasmid DNA isolation kit (Evrogen, Moscow, Russia) according to the manufacturer’s instructions.

All PCRs were conducted using the Encyclo Plus PCR kit (Evrogen, Russia). The primers utilized in this study are detailed in Table 1.

The backbone plasmids pPH7272, a derivative of pPH727-AOH727, and pPA7272 were obtained and described previously [21,22,23]. The other constructs, generated in the present study, are outlined in Table 2.

Table 2 provides an overview of the plasmids that were constructed and employed during the course of this research project.

### 2.2. Yeast Transformation

The heterothallic yeast strains were obtained using *K. kurtzmanii* Y-727 *his4Δ arg4Δ* as the basic strain [27]. Prior to transformation, the integrating DNA fragments were excised from the corresponding plasmids using MluI restriction enzyme, following the purification with a Cleanup S-Cap column kit (Evrogen, Russia) in accordance with the manufacturer’s instructions.

The preparation of competent cells and transformation were conducted in accordance with the methodology described by Kumar and co-authors [28]. Transformants were selected on a YNBD medium, comprising 6.7 g/L yeast nitrogen base (YNB) with ammonium sulfate (Amresco, Solon, OH, USA), 20 g/L glucose (Molekula Ltd., Darlington, UK), and 20 μg/mL histidine (YNBD + his) or arginine (YNBS + arg), depending on the phenotype of the transformants sought.

### 2.3. Isolation of Chromosomal DNA and PCR Amplification of Target Fragments

*K. kurtzmanii* genomic DNA was isolated as described previously [29]. In particular, yeast was cultivated for 18 h in liquid YPD medium (comprising 1% yeast extract, 2% peptone-140, and 2% glucose) on a rotatory shaker at 30 °C and 250 rpm, resulting in an optical density of approximately 10 at 600 nm. Subsequently, cells from 1.5 mL of overnight culture were harvested by centrifugation (5000× *g*, 5 min), resuspended in 700 µL of 1.5 M ammonium acetate solution, and incubated for 10 min at 25 °C. The cells were then harvested by centrifugation (13,000× *g*, 10 min), resuspended in 600 μL of freshly prepared buffer A (1% sodium dodecyl sulfate, 20 mM Tris-HCl pH 8.0, 20 mM EDTA) and incubated for 10 min at 65 °C. Subsequently, 200 μL of a 7.5 M ammonium acetate solution and 100 μL of chloroform were added to the cell suspension, which was then mixed by vortexing and centrifuged at 13,000× *g* for 10 min. The supernatant was transferred to clean tubes and mixed with 600 µL isopropanol. This mixture was then incubated for 10 min at 25 °C, after which DNA precipitation was achieved by centrifugation at 13,000× *g* for 10 min. The DNA precipitate was then washed three times with 70% ethanol and dissolved in 100 µL of deionized water.

The direct PCR amplification of target DNA fragments from yeast colonies was conducted in accordance with a publicly available protocol (https://www.protocols.io/view/yeast-colony-pcr-it-doesn-t-get-any-easier-than-th-e6nvwb82vmkj/v1) (accessed on 5 December 2024).

### 2.4. Construction of the Yeast Strains

For the proof-of-concept experiment for I-SceI meganuclease-based protocols for genetic manipulation of *K. kurtzmanii*, the strain Y-727(sce) possessing *sce* recognition sites flanking *HIS4* gene was generated. This strain was obtained through the transformation of strain Y-727 *his4Δarg4Δ* with an *AOX1::(sce-HIS4-sce)* DNA fragment derived from plasmid pUC19m_(sce), which had been subjected to MluI restriction enzyme treatment. The integration of the *sce-HIS4-sce* cassette was targeted to the distal promoter region of the AOX1 gene, which retained its functionality. The yeast strain Y-727(sce/I-SceI) was generated through the transformation of strain Y-727(sce) with pPA7272_I-SceI plasmid.

For the creation of heterothallic *K. kurtzmanii* strains using developed I-SceI-mediated genetic manipulation protocol, the strains 727-2 and 727-9 were generated by transforming strain Y-727 *his4Δ arg4Δ* with a DNA fragment of plasmid pUC19m_∆MAT, excised from the vector with MluI restriction enzyme. The *sce-HIS4-sce* cassette was integrated into any of the *MAT* loci via gene replacement. Yeast strains 727-2(I-SceI) and 727-9(I-SceI) were obtained through the transformation of plasmid pPA7272_I-SceI into cells from strains 727-2 and 727-9, respectively. The preparation of strains Y-727-2(alpha)-1, Y-727-2(alpha)-2, Y-727-2(alpha)-3, and Y-727-9(a)-1, Y-727-9(a)-2, and Y-727-9(a)-3 is described in the Results and Discussion section.

The pairs of yeast strains, Y-727(His + arg-) and Y-727(Arg + his-), Y-727α(His + arg-) and Y-727α(Arg + his-), Y-727a(His + arg-) and Y-727a(Arg + his-), that were used for strain hybridization analysis, were generated through the transformation of strains Y-727 his4Δ arg4Δ, Y727-2(alpha)-1 and Y727-9(a)-1 with plasmids pPH7272_βgal and pPA7272_βgal, respectively.

**Figure 1:**
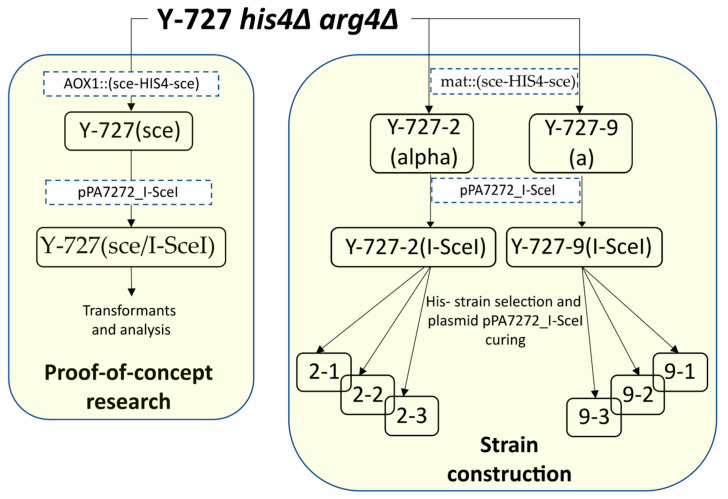
Scheme of construction of the main strains. The strains are listed in Table 3. Transformation steps are indicated by dotted brackets with the constructs used.

The strains used in this work are outlined in Table 3 and the strain development history is shown in Figure 1.

### 2.5. The I-SceI-Mediated Elimination of the HIS4 Gene

The cells from the recipient strains of *K. kurtzmanii* containing the integrated *sce-HIS4-sce* cassette were transformed with the episomal plasmid pPA7272_I-SceI by electroporation, as described by Kumar [28]. The electroporated cells were immediately washed with a sterile, ice-cold 1M sorbitol solution and centrifuged at 13,000 rpm for 30 s. The supernatant was then removed. The precipitated cells were then resuspended in 3mL of YPD, followed by culturing using an orbital shaker (250 rpm) at 30 °C for three hours. Following the recovery phase, the cells were harvested by centrifugation (13,000 rpm, 30 s) and the synthesis of I-SceI meganuclease was induced by resuspension of cells in 3 mL of YPM medium (1% yeast extract, 2% peptone, and 1% methanol) and cultivation at 30 °C and 250 rpm for 18 h. The induced cells were harvested by centrifugation (13,000× *g*, 30 s), resuspended in 20 mM PBS (pH 7.4) and plated on selective YNBD + his medium containing 30 mg/L histidine. In the control experiment, the transformed cells were cultured without methanol induction.

### 2.6. Elimination of pPA7272_I-SceI Plasmid

To eliminate the pPA7272_I-SceI plasmid from yeast cells, a routine plasmid curing procedure based on spontaneous ARS plasmid elimination was used. For this purpose, the corresponding transformants containing the pPA7272_I-SceI plasmid were grown under non-selective conditions for about 10 generations and the resulting cultures were plated on non-selective agar. The resulting clones were screened for the *arg-* phenotype.

### 2.7. Hybridization of Strains and Treatment of Spores with Diethyl Ether

The hybridization of strains was conducted in accordance with a previously published procedure [31]. Briefly, fresh cultures of the strains were plated in parallel strokes on YPD dishes and incubated for 24 h at 30 °C. The cells of the hybridization partner strains were crossed by printing at an angle of 90° on a dish containing SporeN sporulation medium (0.5% sodium acetate, 1% KCl, 1% glucose, 2% agar) and incubated for a period of three days at a temperature of 25 °C. Following this, the cells were transferred by printing to a YNBD dish in order to identify the diploid hybrids that had formed.

To induce spore formation, fresh cell cultures were seeded on SporeN and incubated for seven days at 25 °C. Spore extraction was performed using diethyl ether in accordance with the protocol for random spore analysis [31].

### 2.8. Analysis of Recombinant β-Galactosidase

Yeast cells transformed with plasmid pPH7272_βgal, which provided synthesis of secreted β-galactosidase, were grown on YPGM medium (1% yeast extract, 2% peptone-140, 0.5% glycerol, 1% methanol) using an orbital shaker (250 rpm) at 30 °C for 72 h. Every 24 h, methanol was added to the medium to a final concentration of 1% (vol/vol). At the end of the cultivation, the optical density of the culture was measured (OD600), the cells were precipitated by centrifugation, and a sample of the supernatant was collected for the measurement of β-galactosidase activity and the SDS-PAGE assay.

The β-galactosidase activity was quantified at 43 °C in accordance with the following methodology. A total of 850 μL of 100 mM potassium phosphate buffer at pH 6.5 and 100 μL of 0.05 M aqueous ONPG solution (Thermo Scientific, Foster City, CA, USA) were added to the tube, mixed, and heated at 43 °C for 2 min. A 50 μL sample of the culture fluid, diluted at least 10-fold in 100 mM potassium phosphate buffer at pH 6.5, was then added to the warmed reaction mixture. The reaction was terminated by the addition of 500 µL of 1 M sodium carbonate solution. The results were analyzed by measuring the absorbance of the reaction mixture at λ = 420 nm. The amount of enzyme required to form 1 μM reaction product (ONP) in 1 min at 43 °C in 100 mM potassium phosphate buffer pH 6.5 was taken as a unit of activity. The molar absorption coefficient of ONP was set at 4500 M^−1^ cm^−1^ in the calculations.

### 2.9. Whole-Genome Sequencing, De Novo Assembly and Genome Analysis

High molecular weight genomic DNA was isolated from 20 mL of overnight culture in YPD medium in accordance with the protocols recommended by Oxford Nanopore Technologies [32]. Subsequently, the isolated DNA was subjected to further re-purification using the QIAGEN Genomic-tip 20/G kit (Qiagen, Hilden, Germany). The quality of the isolated DNA was evaluated spectrophotometrically on a Nanodrop 1000 device (Thermo Fisher Scientific, Waltham, MA, USA) by calculating the A260/A280 and A260/A230 absorbance ratios. The DNA concentration was determined using a Qubit 4.0 fluorimeter with the Qubit dsDNA BR Assay Kit (both—Thermo Fisher Scientific, Waltham, MA, USA). The level of nucleic acid degradation was evaluated through the analysis of a 0.8% agarose gel prepared on a Tris-acetate buffer (1× TAE) containing ethidium bromide (0.1 μg/mL).

The samples were sequenced on a GridION sequencer using the Rapid Barcoding Sequencing Kit (art. SQK-RBK004) on a FLO-MIN106D cell (Oxford Nanopore Technologies, Oxford, UK), in accordance with the manufacturer’s instructions.

Trimming of sequencing adapters and quality filtering was performed with Porechop [33]. The genome assembly was conducted using the Canu 2.2 software with –*nanopore-raw* and *genomeSize = 9.2 m* settings [34]. A whole-genome alignment was conducted using the NUCmer 3.1 software [35]. The prediction of protein-coding genes was conducted using GeneMark-EP + 4.72 [36], while the functional annotation of the predicted proteins was performed using EggNOG-mapper 2.1.12 [37]. A search for homologous genome regions and protein-coding sequences was conducted using the NCBI BLAST+ web interface [38]. Additional structural and functional annotations were performed with the Yeast Genome Annotation Pipeline web server [39]. Alternative de novo assembly used for the in silico confirmation of chromosome fusion (described in Section 3.4) was performed using Flye v.2.9.3 with parameters *–nano-raw* (type of input reads) and *-i 3* (number of polishing iterations) [40]. Analysis and visualization of read mapping in the chromosome fusion region were performed with CLC Genomics Workbench v.24.0.1 using the ‘Map Long Reads to Reference’ tool with default parameters. Porechop-trimmed reads were used for mapping.

### 2.10. Pulsed-Field Gel Electrophoresis (CHEF)

Intact chromosomal DNAs were obtained as described previously [41]. A CHEF-DR III apparatus (Bio-Rad, Hercules, CA, USA) was used to separate the chromosomal DNAs. The electrophoresis buffer was 0.5× TBE (45 mM TrisHCl, pH 8.0, 10 mM EDTA, 45 mM boric acid). The buffer was circulated around the 0.9% gel and cooled to 15 °С. The following three-step program was used for electrophoresis: (1) 150 V for 10 h with a switching time of 40–120 s (linear ramping); (2) 130 V for 28 h with a switching time of 120–360 s (linear ramping); and (3) 100 V for 10 h with a switching time of 360–1200 s (linear ramping). The chromosome sizes of the strains studied were estimated using commercial standards *Saccharomyces cerevisiae* YNN 295 and *Wickerhamomyces canadensis* YB-4662-VIA (Bio-Rad, Hercules, CA, USA). After electrophoresis, the gel was stained with ethidium bromide to visualize the chromosome bands.

## 3. Results

### 3.1. Development of I-SceI Meganuclease-Based Protocols for Genetic Manipulation of K. kurtzmanii

A review of the available data indicates that the genome of *K. kurtzmanii* lacks the sequence TAGGGATAACAGGGTAAT, which is the specific recognition site for the I-SceI meganuclease [7,24,42]. This paves the way for the convenient and reliable utilization of I-SceI in *K. kurtzmanii* cells.

The development of efficient techniques for using I-SceI to edit the genome of *K. kurtzmanii* was successfully completed. This was achieved on the basis of the recipient strain Y-727 his4Δ arg4Δ (Table 3). An integrative DNA fragment, designated AOX1::(*sce-HIS4-sce*), was constructed. This fragment contains the HIS4 gene of *K. kurtzmanii*, and it is surrounded by the recognition sites of the I-SceI restriction enzyme (sce). The structure of this fragment is illustrated in Supplementary Figure S1A. The fragment was integrated into the promoter region of the AOX1 gene of *K. kurtzmanii*, resulting in the creation of a new strain, designated Y-727(sce). The integration of the fragment into the AOX1 gene promoter was confirmed by PCR (Supplementary Figure S3).

To eliminate the HIS4 construct from the *AOX1* locus, the obtained strain Y-727(*sce*) was transformed by the episomal plasmid pPA7272_I-SceI. This plasmid provided the synthesis of the I-SceI meganuclease, which was under the control of the P*_FDH_* promoter (Supplementary Figure S1B).

Two protocols of I-SceI meganuclease induction in transformed cells were compared in order to ascertain the most efficacious method. In the initial variant (Method 1), I-SceI synthesis was initiated directly following transformation, without the intermediate selection of transformants, as detailed in the Materials and Methods section. In the second variant (Method 2), the transformation was conducted on colonies on selective medium without arginine. Subsequently, the synthesis of I-SceI meganuclease was induced in the cells of the selected transformants. The induction and re-selection of Arg+ transformants were carried out under the same conditions as in Method 1.

In the control experiments, glucose was introduced to the medium for the cultivation of transformants in place of the methanol inducer, which resulted in P_FDH_ repression [43] and thus impeded the synthesis of meganuclease.

The elimination of HIS4 by meganuclease was monitored by determining the ability of cells to synthesize histidine. The fraction of cells that lost the ability to synthesize histidine was determined by reprinting transformants subjected to methanol induction by Method 1 or Method 2 onto dishes with selective medium without histidine. The results of this analysis are presented in Table 4.

The results obtained were contrary to expectations: auxotrophic strains of his- were only detected as a consequence of induction by Method 1. These transformants constituted approximately 75% of the total number of transformants, indicating that the meganuclease I-SceI exhibited high functional activity in *K. kurtzmanii* cells. Conversely, no auxotrophic strains of his- were detected among the transformants obtained by Method 2 (Table 4).

The results obtained indicated the possibility of efficient use of the meganuclease I-SceI for genetic manipulations in *K. kurtzmanii* cells. Furthermore, the necessity of I-SceI induction directly in freshly transformed cells was established.

### 3.2. Production of Heterothallic Strains of K. kurtzmanii

It is established that the inactivation or elimination of any of the MAT alleles (MAT**a**1/MAT**a**2 or MATα1/MATα2) results in loss of self-diploidisation and subsequent sporulation. These strains are therefore carriers of a heterothallic phenotype [25]. Additionally, heterothallic strains can be generated through the inactivation of the chromosomal inversion mechanism [24].

**Figure 2:**
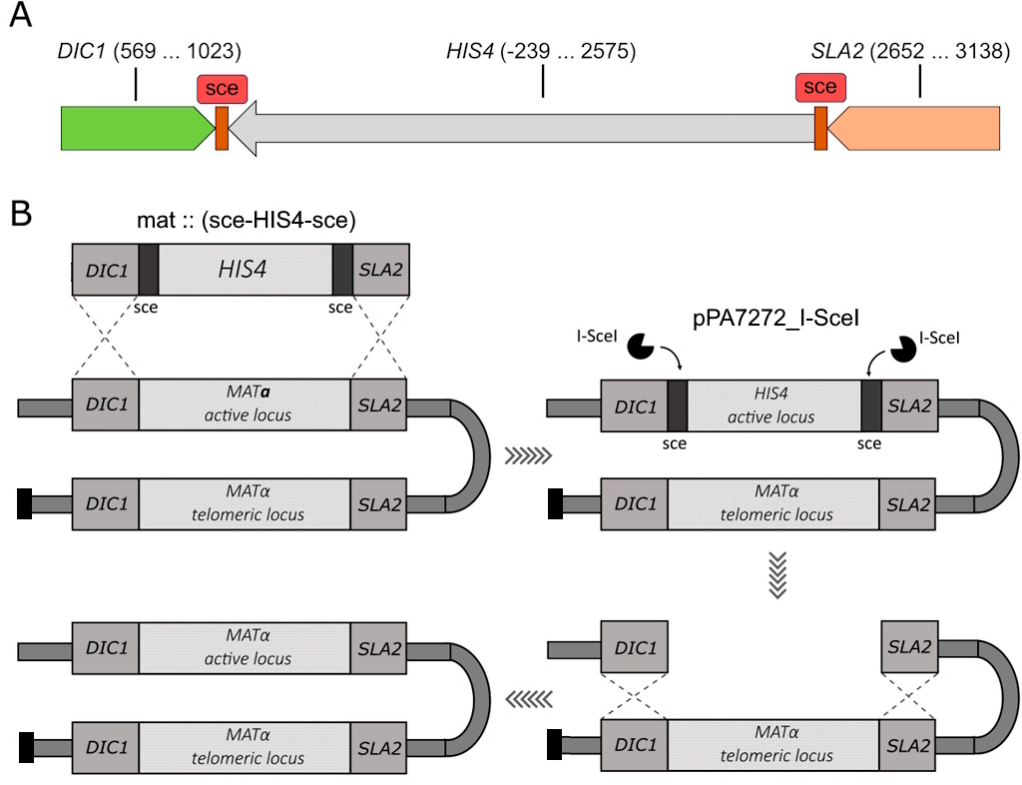
(**A**). Schematic of the sce-HIS4-sce DNA fragment designed for integration into the *K. kurtzmanii* genome. The DIC1 and SLA2 homology regions, which guide the integration of the target fragment into MAT loci, are shown. The DNA end nucleotide positions of the cloned DIC1, SLA1 and HIS4 genes are given in brackets. For each gene, the positions are given relative to the corresponding ATG(+1) start codon; (**B**). Schematic of the production of heterothallic yeast strains of *K. kurtzmanii* (see explanations in the text).

To address the same issue in *K. kurtzmanii*, we opted for an alternative approach that entailed minimal alterations to the yeast genome while maintaining the integrity of the MAT loci and inversion mechanism (Figure 2B). Unifying the contents of the *MAT* loci should ensure the absence of mating-type switch. To this end, a double-stranded break was to be made at either of the two loci, active or silent, in order to take advantage of the homologous flanking sequences of the loci and activate the repair mechanism by homologous recombination. The I-SceI meganuclease was selected for introduction of a site-specific double-stranded break at the MAT locus.

In order to integrate I-SceI recognition sites into the yeast MAT locus, a plasmid pUC19m_∆MAT was constructed, containing the target cassette mat::(sce-HIS4-sce) (Figure 2A). The homology regions included DIC1 and SLA2 gene sequences of approximately 300 base pairs in length from the natural environment of the MAT loci mating-type genes. The selectable HIS4 gene, surrounded by I-SceI meganuclease recognition sites, was integrated into the MAT loci of yeast *K. kurtzmanii* using the substitution method.

**Figure 3:**
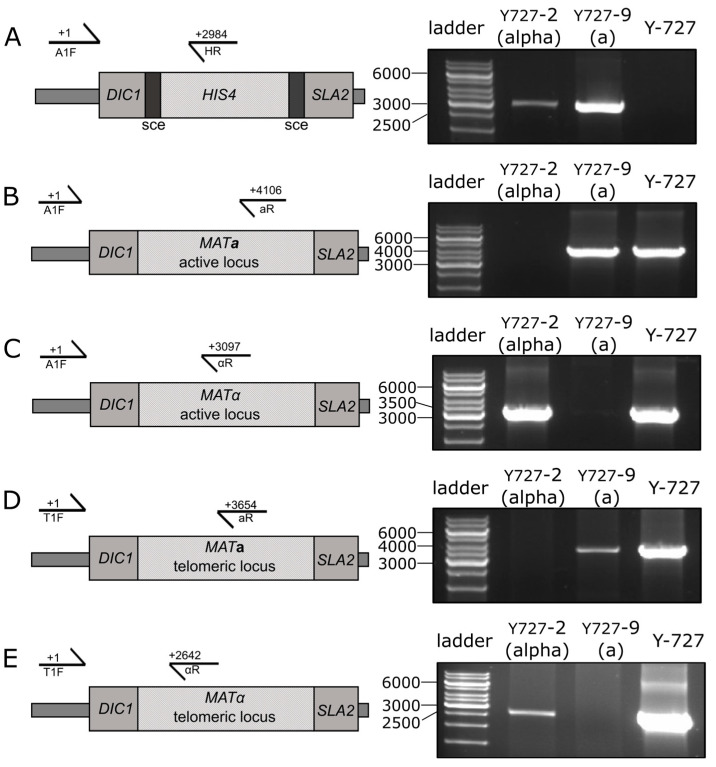
PCR analysis of the MAT loci of the chromosomal DNA of strains Y727-2(alpha), Y727-9(a) and the parental control strain Y-727 his4Δ arg4Δ of *K. kurtzmanii*. (**A**) Analysis of the integration of the sce-HIS4-sce cassette into the MAT locus of the yeast genome. (**B**–**E**), Analysis of the structure of the active (**B**,**C**) and telomeric (**D**,**E**) MAT loci of yeast strains. Schematics of the yeast active and telomeric MAT loci (left) and PCR amplification results (right) are shown. Amplification was performed using primers A1F, T1F, aR, αR and HR (Table 1). The positions corresponding to the 5′ end residues of the primers are indicated in the figure. GeneRuler 1 kb DNA Ladder (Thermo Fisher Scientific, Waltham, MA, USA) was used as the DNA ladder. Original images can be found in Figures S8 and S9.

The recipient strain, *K. kurtzmanii* Y-727 his4Δ arg4Δ, was transformed using the mat:::(sce-HIS4-sce) cassette, resulting in the generation of a collection of Y-727(sce) strains. Since the potential characteristics of heterothallic strains were not known in advance, two strains with different mating types were specifically selected using PCR analysis. The cells of strains Y-727-2(alpha) and Y-727-9(a) contained an integrated cassette (Figure 3A), and its integration resulted in the substitution/inactivation of MATα and MAT**a** alleles, respectively, without affecting the alternative alleles of MAT genes (Figure 3B–E). At the same time, the data demonstrated that strains Y-727-2(alpha) and Y-727-9(a) retained the capacity for chromosomal inversions, as evidenced by the presence of intact, corresponding MATα and MAT**a** genes in their cells at both the active (Figure 3B,C) and telomeric loci (Figure 3D,E).

The PCR data unambiguously demonstrated that the obtained clones Y727-2(alpha) and Y727-9(a) represented the desired heterothallic strains of mating type α and **a**, respectively. However, despite the heterothallic status, cultures of both strains retained a heterogeneous composition. This was due to chromosomal inversions that altered the localization of the remaining intact MATα or MAT**a** genes and the sce-HIS4-sce cassette at the active or telomeric loci. As a result, the cells of these strains may or may not exhibit a mating type.

### 3.3. Elimination of the sce-HIS4-sce Cassette Using the I-SceI Meganuclease

Monocultures of heterothallic strains Y727-2(alpha) and Y727-9(a) were obtained by means of site-directed double-stranded hydrolysis of sce-HIS4-sce DNA that had been integrated into the MAT locus. It was hypothesized that repair of this break via the mechanism of homologous recombination would result in the restoration of the inactivated locus, while unifying the contents of both MAT loci. We anticipated that the utilization of two sce sites would facilitate the elimination of HIS4 and enhance the yield of the desired strains. The function of matrices in this process was attributed to intact MATα and MAT**a** alleles, which were retained in the genomes of strains Y727-2(alpha) and Y727-9(a) surrounded by DIC1 and SLA genes that replicated the environment of the sce-HIS4-sce cassette (Figure 2B).

Double-strand breaks were introduced in the sce sites of the integrated cassette by transforming strains Y727-2(alpha) and Y727-9(a) cells with plasmid pPA7272_I-SceI, which provides meganuclease synthesis. The synthesis of the enzyme in the cells of the obtained transformants was induced in accordance with the protocol established in the preceding section of the work. Intact strains Y727-2(alpha) and Y727-9(a), as well as transformed but uninduced cultures of these strains, served as controls.

The results demonstrated that the elimination of the HIS4 gene integrated into the MAT loci of strains Y727-2(alpha) and Y727-9(a) was contingent upon the synthesis of meganuclease I-SceI. The methanol induction of the original, untransformed strains Y727-2(alpha) and Y727-9(a) did not result in the appearance of auxotrophic *his-* colonies (Table 5), indicating high stability of the integrated cassette. Concurrently, the induction of I-SceI synthesis in Y727-2(alpha) and Y727-9(a) cells transformed with plasmid pPA7272_I-SceI resulted in the emergence of a considerable number of *his-* clones (Table 5). Furthermore, the high efficiency of HIS4 elimination from the MAT loci (60% and 74%, Table 5) was comparable to the previously obtained value for the process at the AOX1 locus (76%, Table 4).

Given the lack of control over yeast genome repair processes, three independently derived derivatives of strains Y727-2(alpha) and Y727-9(a), which exhibited the desired *his-* phenotype, were selected for further analysis. Subsequently, the plasmid pPA7272_I-SceI was eliminated from the cells of the selected transformants, resulting in the designation of the resulting plasmid-free strains with the *his-arg-* phenotype as Y727-2(alpha)-1, Y727-2(alpha)-2, Y727-2(alpha)-3 and Y727-9(a)-1, Y727-9(a)-2, and Y727-9(a)-3, respectively.

### 3.4. Genome Analysis of Heterothallic Derivatives of Y727 his4Δ arg4Δ

Primary analysis of rearrangements in final hetherothallic strains, namely Y727-2(alpha)-1,2,3 and Y727-9(a)-1,2,3, was performed using PCR as described in Supplementary Text. Results confirmed desired unification (duplication) of MATa genes in the active and telomeric MAT loci of strains Y727-9(a)-1,2,3. At the same time, the amplification results of the telomeric MAT locus in strains Y727-2(alpha)-1,2,3 were not as expected (Supplementary Text, Supplementary Figure S6). Therefore, for comparative analysis of chromosomal rearrangements, the strains Y727-2(alpha)-1, Y727-9(a)-1 and the parental strain Y727 *his4Δ arg4Δ* were sequenced using Oxford Nanopore long-read sequencing technology.

Nanopore sequencing and primary read filtering performed with porechop [33] resulted in 117, 111 and 149 thousands of long reads with N50 of 20.5, 13.7 and 17.5 kb for strains Y727, Y727-9(a)-1 and Y727-2(alpha)-1, respectively. The de novo assembly of genomes using Сanu assembler [34] yielded three-to-four long contigs with the expected coverage. Contigs with significant variation in coverage were multiple amplified copies of mitochondrial DNA or rDNA (Supplementary Table S1). The lengths of assembled chromosomes compared with the only RefSeq genome sequence of the genus Komagataella (*K. phaffii*, GCF_000027005.1) and with the most complete Komagataella genome, described by Sturmberger et al. [14], are presented in Table 6.

**Figure 4:**
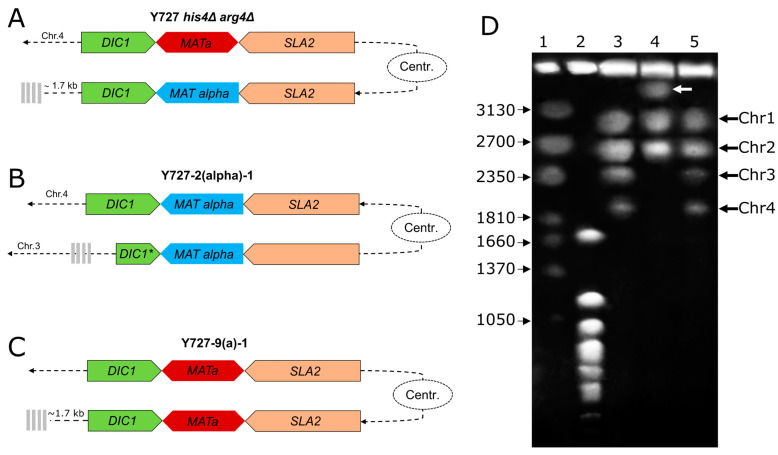
Structure of chromosomes containing MAT loci of Y727 his4Δ arg4Δ and its heterothallic derivatives. (**A**). Structure of MAT loci in the strain Y727 his4Δ arg4Δ. (**B**). Structure of MAT loci in the strain Y727-2(alpha)-1. (**C**). Structure of MAT loci in the strain Y727-9(a)-1. The MAT loci genes are indicated by colored pentagons, the centromere is represented by an oval, and telomeres are shown by gray vertical bars. (**D**) Karyotype analysis of chromosomal DNAs of *K. kurtzmanii* strain Y-727 his4Δ arg4Δ and its derivatives. 3–Y-727 his4Δ arg4Δ; 4–Y727-2(alpha)-1; and 5–Y727-9(a)-1. Chromosome standards: 1—Wickerhamomyces canadensis YB-4662-VIA; 2—Saccharomyces cerevisiae YNN 295; and the chromosome sizes (kb) of the standard strains are indicated to the left. White arrow indicates the chromosomal band of ca. 4000 kb in strain Y727-2(alpha)-1. Original images can be found in Figure S10.

In the case of strain Y727-2(alpha)-1, a large contig with a length of 4.12 Mbp was identified as representing a fusion of chromosomes 3 and 4 of the original strain (Figure 4B, Supplementary Figure S6D). Chromosomes were fused by their short arms, with one of the chromosomes being in the opposite direction compared to the parental strain. Further coverage analysis of the fusion region of sequences belonging to chromosomes 3 and 4 revealed no evidence of misassemblies. Additionally, comparative analysis of this assembly with the assembly obtained with the Flye assembler [40] also confirmed chromosomal rearrangement.

To provide experimental confirmation, primer T2F was designed to the subtelomeric region of chromosome 3. Following PCR with primer TA1R annealing to the SLA2 gene and subsequent digestion of the amplicon by EcoRV/MluI endonucleases, the expected fragments were yielded, with lengths of 2025, 1602, 807, and 587 bp, as anticipated for all heterothallic strains, Y727-2(alpha)-1,2,3 (Supplementary Text, Supplementary Figure S4F).

Further confirmation of the chromosome fusion was obtained using pulsed-field gel electrophoresis (Figure 4D). Initial strain Y-727 *his4Δ arg4Δ* and its derivative Y727-9(a)-1 showed identical karyotypes with four chromosome bands (lanes 3 and 5). In contrast, strain Y727-2(alpha)-1 displayed a notably different pattern with three chromosome bands (lane 2). Instead of the two missing smallest chromosomes 3 and 4, there was a large chromosome of about 4000 kb, which appears to be a fused dicentric chromosome.

The local structure of the MAT loci was found to be largely consistent with expectations. In strain Y727-9(a)-1, two MAT loci situated at the identical distance from the centromere as in the parental strain exhibited the presence of two copies of the MAT**a**1, MAT**a**2, DIC1, and SLA2 genes, each (Figure 4D). In contrast, strain 2-1 exhibited a partial deletion of the DIC1 gene resulting from the inversion of a substantial portion of chromosome 4, including the MAT loci and centromere. This event did not affect the integrity of the MATα1, MATα2, and SLA2 genes (Figure 4A).

### 3.5. Strain Hybridization Analysis

The capacity of the parent strain Y727 his4Δ arg4Δ, in addition to its heterothallic derivatives Y727-2(alpha)-1 and Y727-9(a)*-1*, to undergo self-diploidization and hybridize was assessed through the implementation of two distinct methodologies.

The process of self-diploidization was initiated by incubating the strains on nitrogen-free SporeN medium, as detailed in “Section 2”. The induced strain cultures were treated with diethyl ether and seeded onto dishes with nonselective medium. This procedure ensured the death of vegetative cells, leaving only spores, which were the progeny of strains capable of self-diploidization with the formation of fertile diploids, viable [31].

The results demonstrated that the control strains that were not treated with diethyl ether, as well as the initial parental strain Y727 his4Δ arg4Δ that was treated with ether, exhibited high survival rates under the experimental conditions (Table 7). Conversely, the application of diethyl ether to strains Y727-2(alpha)-1 and Y727-9(a)-1 resulted in the complete loss of their viability. Therefore, the results obtained corroborate the hypothesis that strains Y727-2(alpha)-1 and Y727-9(a)-1 are unable to undergo self-diploidization and subsequent spore formation.

In order to perform a comprehensive analysis of the hybridization potential of the strains Y-727 his4Δ arg4Δ, Y727-2(alpha)-1 and Y727-9(a)-1, we obtained their derivatives, in which the auxotrophic mutations his4Δ and arg4Δ were complemented. For this purpose, the laboratory plasmids pPH7272_βgal and pPA7273_βgal containing the HIS4 and ARG4 genes were integrated into the cells of the aforementioned strains. This resulted in the generation of paired derivatives, Y-727(His + arg-), Y-727(Arg + his-), Y727-2(alpha)-1 (His + arg), Y727-2(alpha)-1 (Arg + his-), Y727-9(a)-1 (His + arg-) and Y727-9(a)-1 (Arg + his-) with differences in the set of auxotrophic mutations (Table 1). Hybridization of these strains allowed for the evaluation of hybrid growth on appropriate selective media.

The outcomes of the crossing and growth tests are presented in Table 8 and Supplementary Figure S4. The data obtained substantiated the hypothesis that the original parental strain, Y-727 his4Δ arg4Δ, is capable of efficient hybridization. The paired derivatives exhibited the capacity to cross successfully with both one another and with the corresponding derivatives of strains Y727-2(alpha)-1 and Y727-9(a)-1 (Table 8). Conversely, hybridization between paired strains Y727-2(alpha)-1 (His + arg-) and Y727-2(alpha)-1 (Arg + his-), as well as Y727-9(a)-1(His + arg-) and Y727-9(a)-1(Arg + his-), was found to be entirely ineffective, as these pairs were unable to form prototrophic diploids (Table 8, Supplementary Figure S5). Nevertheless, the crossbreeding of these strains in various combinations, both with each other and with derivatives of the parental strain, revealed that the hybridization ability of strains Y727-2(alpha)-1 and Y727-9(a)-1 was completely conserved (Table 8, Supplementary Figure S7).

The results were therefore definitive in indicating that strains Y727-2(alpha)-1 and Y727-9(a)-1 had retained the capacity for efficient hybridization but had lost the capacity for self-diploidization. The experiments thus unambiguously proved the heterothallic status of the obtained strains Y727-2(alpha)-1 and Y727-9(a)-1.

### 3.6. Evaluation of Secretory Activity of Producers Based on Strains Y-727-2(alpha) and Y-727-9(a)

As mentioned above, the production of heterothallic strains of *K. kurtzmanii* was aimed at improving the stability of future producer strains. In this regard, a small comparative study was performed to evaluate the potential effect of genetic modifications of derivatives of the strains Y727-2(alpha) and Y727-9(a) on their secretory activity. The strains Y727-2(alpha)-1,2,3 and Y727-9(a)-1,2,3 and the parental control strain Y-727 his4Δ arg4Δ were transformed with the vector pPH7272_βgal (Supplementary Figure S1), which contains the β-galactosidase gene of Aspergillus oryzae under the control of the AOX1 promoter of *K. kurtzmanii*. The secretion of the target protein was directed by the standard α-factor leader region of the yeast *S. cerevisiae*. Three independently derived transformants were selected for analysis of target protein production by each strain tested. Reporter protein synthesis was performed under methanol induction conditions.

**Figure 5:**
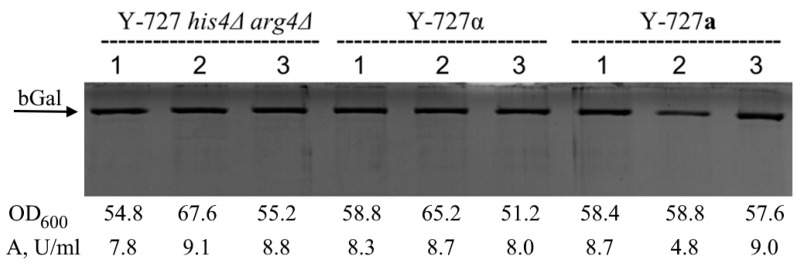
Comparative analysis of the secretory activity of strains Y-727 his4Δ arg4Δ, Y727-2(alpha)-1,2,3 and Y727-9(a)-1,2,3 transformed with vector pPH7272_βgal (producers of recombinant β-galactosidase of *A. oryzae*). Transformants were cultivated in Erlenmeyer flasks with a total volume of 750 mL and each contained 50 mL of YP growth medium supplemented with methanol to 1% every 24 h for induction. Cultivation was carried out before the stationary phase using a rotatory shaker with mixing speed 250 min^−1^ at 30 °C for 72 h. Ten μL of supernatants were applied to the electrophoresis. The optical density of cultures (OD_600_) and β-galactosidase activity in supernatants of the corresponding transformants are presented below the electrophoregram. Original images can be found in Figure S11.

The results of the analysis (Figure 5) demonstrated that the transformed derivatives of genetically modified strains Y727-2(alpha)-1,2,3 and Y727-9(a)-1,2,3 **a**, as well as the original parental strain, exhibited a comparable level of β-galactosidase synthesis. Consequently, the findings suggested that the genetic modifications did not influence the capacity of the obtained heterothallic strains Y727-2(alpha)-1,2,3 and Y727-9(a)-1,2,3 to effectively secrete protein.

## 4. Discussion

### 4.1. Development of a Novel Genetic Tool for the Protein Production Platform Based on Non-Conventional Yeast K. kurtzmanii

The advantages of non-conventional yeasts, in particular their tolerance to unfavorable conditions and their ability to grow on inexpensive substrates, make them a promising option for biotechnological applications [44]. *Komagataella kurtzmanii* expression systems provide an efficient alternative to the widely used commercial systems of *Komagataella phaffii* (formerly *Pichia pastoris*) [30]. They offer certain advantages in the expression and secretion of recombinant proteins as well as in the production of industrially important enzyme preparations [22].

The creation of novel platforms for heterologous expression necessitates the development of efficient genetic tools. One particularly prevalent genetic tool employed for the manipulation of both prokaryotic and eukaryotic genomes is the I-SceI homing endonuclease [9]. The use of the I-Sce system has been described in a number of different contexts, including mammalian cell cultures [45], *D. melanogaster* [46], prokaryotic microorganisms [20], mycelial fungi [17] and the baker’s yeast *S. cerevisiae* [15]. However, to date, there have been no reports of its use in members of the genus *Komagataella*. In this study, we successfully employed the I-Sce system to edit the genome of *K. kurtzmanii* via transient expression of I-Sce under the control of a methanol-induced P*_FDH_* promoter [47].

However, experiments to optimize the transformation protocol demonstrated that when an additional selection of clones carrying plasmid pPA7272_I-SceI using a selective medium without arginine is employed, clones in which the target gene is deleted using I-Sce are not identified. Concurrently, induction with methanol immediately following transformation yields a positive result. This may be attributed to the partial ’toxicity’ of prolonged expression of meganuclease I-SceI in yeast cells and its ability to act as a negative strain selection factor in this regard. A prerequisite for the constitutive synthesis of I-SceI is the activity of the P*_FDH_* promoter, which controls its expression. In contrast to another methanol-inducible promoter P*_AOX1_* in the closely related yeast *K. phaffii*, the P*_FDH_* promoter is known to retain a significant level of activity on glucose medium [47]. While studies analyzing the toxicity of I-Sce in mammalian cells demonstrate its almost complete absence in comparison to other homing endonucleases [48], this observation necessitates further analysis of Sce toxicity with respect to *Komagataella*.

### 4.2. Generation of New Heterothallic Strains of Komagataella kurtzmanii

The yeast *K. kurtzmanii* and other pre-whole genome duplication yeasts possess an ancient mechanism of mating-type regulation. This mechanism is activated by nitrogen starvation and/or on a glucose-containing medium. [6,49]. In *K. phaffi*, the mating-type switch is achieved not through gene conversion as observed in baker’s yeast, but rather through inversion of the chromosome section of the chromosome section including the MAT loci at its termini [3]. This results in a shift of a (alpha) genes to the repressed, subtelomeric region and of alpha (a) genes to the actively expressed, euchromatin section of the chromosome. It seems reasonable to posit that *K. kurtzmanii* employs a similar mechanism. Consequently***,** K. kurtzmanii* cultures consist of a heterogeneous population of cells with varying mating types, which can undergo spontaneous and undesirable crossing and sporulation. This introduces an additional layer of genetic instability into the cell population, potentially limiting their suitability for industrial applications since one of the crucial requirements for industrial use is the homogeneity of all cells within the system. To create stable heterothallic *K. phaffii* strains, Heistinger and colleagues implemented the deletion of the outer region of the MAT locus containing the *DIC1* gene. That led to the inactivation of the homologous recombination mechanisms and therefore led to the block of mating-type switching [25]. An alternative approach was selected to achieve this goal. Rather than blocking the mechanism of cell mating-type switch, the unification of MAT loci content was employed to prevent mating-type change. We considered the main advantage of the unification approach over the inactivation approach to be the maximum preservation of all genetic mechanisms characteristic of natural yeast strains (it cannot be excluded that the mechanism of chromosomal inversions has a pleiotropic effect beyond the switching of cell mating type). Our approach resulted in the production of stable heterothallic strains of *K. kurtzmanii*, which can be utilized for the further development of a platform for recombinant protein expression.

### 4.3. The Non-Homologous End Joining Repair Pathway Is Activated Through the Double-Strand Breaks Introduced by the I-SceI Homing Endonuclease

Due to its extended and therefore rare recognition site, I-SceI homing endonuclease is an effective tool for introducing inherited changes into the genome of model organisms with high accuracy [50]. However, they introduce cytotoxic double-strand breaks that must be repaired via one of two mechanisms: non-homologous end joining (NHEJ), which involves direct ligation of free DNA termini, and homologous recombination, which is accomplished by processing the ends of the break and subsequent recombination with a homologous matrix [51]. Although the latter mechanism appears to be more predictable, less error-prone, and consequently less likely to result in functional mutations, non-homologous end joining (NHEJ) is the dominant double-strand break repair pathway in eukaryotic cells [52]. The hypothesis of our experiment was that the deletion of one of the endogenous copies of the MAT locus by I-SceI homing endonuclease would result in the activation of the homologous repair pathway and the insertion of sequences from the second MAT locus. This would lead to the formation of two identical copies of the MAT locus in the genome, and consequently, the resulting strains would be unable to undergo a mating-type switch, although they would fully retain their natural ability to undergo chromosomal inversions. For the Y727-2(alpha)-1 strain, however, it is evident that the recombination process involved the non-homologous end joining (NHEJ) mechanism, resulting in the deletion of most of the DIC1 gene, which is a key participant in the homologous recombination mechanism, and the fusion of chromosomes 3 and 4 of the parental strain. At the same time, in this strain we found an expected rearrangement of the MAT loci and a large inversion of the region between the mating type (MAT) loci, both of which clearly indicate the activity of homologous recombination. Conversely, inspection of the sequences of the MAT loci in strain Y727-9(a)-1 suggests that the non-homologous end joining (NHEJ) mechanism may not be consistently engaged and that homologous recombination was the sole mechanism that led to their rearrangement. It should also be noted that strains Y727-2(alpha)-1 and Y727-9(a)-1 were not exclusive isolates. In both cases, independently generated mutant strains Y727-2(alpha)-1, Y727-2(alpha)-2, Y727-2(alpha)-3, Y727-9(a)-1, Y727-9(a)-2, and Y727-9(a)-3 were selected that had identical PCR analysis patterns within the corresponding triples. Thus, it appears that the complexity of the above circumstances does not allow us to give a consistent explanation.

Nevertheless, to make an attempt to resolve these contradictions, we assumed that the clue to the genetic features of strains Y727-2(alpha)-1 originated in the genome of the intermediate strain Y727-2(alpha). That is, the genetic rearrangements that were subsequently found in strain Y727-2(alpha)-1 first appeared at the stage of integration of the *mat::(sce-HIS4-sce)* cassette into the genome of strain Y-727 *his4Δ arg4Δ*. We believe that integration of the *mat::(sce-HIS4-sce)* cassette into the telomeric regions of MAT provoked a DIC1 partial deletion and chromosome fusion in strain Y727-2(alpha). It is possible that the above changes appeared as a response to the loss of the integrity of the telomeric region of chromosome 4 that accompanied the cassette integration process. At the same time, the integration process itself was probably carried out differently in strains Y727-2(alpha) and Y727-9(a). In the first case, successful recombination affected only the SLA2 arm, resulting in the displacement of the native telomeric end of the chromosome and replacement with an integrative *mat::(sce-HIS4-sce)* fragment. Following this, the DIC1 arm of the integrative fragment underwent trimming and fusion with chromosome 3.

Thus, we believe the strain Y727-2(alpha) acquired the major genetic alterations identified subsequently at the genomic sequencing stage. Subsequently, at the stage of transformation of strains Y727-2(alpha) and Y727-9(a) with a plasmid containing the meganuclease gene, induction of this gene, and introduction of double-stranded breaks, as expected, we activated the most precise mechanism of homologous recombination. It worked without surprises and produced transformants from which we selected strains Y727-2(alpha)-1,2,3 and Y727-9(a)-1,2,3, which contained unified MAT loci and resembled each other as clones of the two prototypic strains. Thus, we believe that although our results look alarming, in reality they prove the efficiency and safety of meganuclease utilization in yeast cells. It is also noteworthy that the genetic manipulations and NHEJ-mediated chromosomal rearrangements in strains Y727-2(alpha)-1 and Y727-9(a)-1 did not result in a notable impact on the secretory activity of producers. This observation suggests that the functional integrity of the genome remained intact.

## 5. Conclusions

The present work addresses the practical problem of obtaining heterothallic strains of *K. kurtzmanii*, which are expected to be more stable and therefore more efficient in technological applications. Therefore, the practical result of this work was the successful generation of heterothallic strains of the yeast *K. kurtzmanii*, which have retained their full secretory potential and are suitable for further biotechnological applications. However, the results of genome sequencing and pulse-field gel electrophoresis unexpectedly revealed that although the problem was solved, the integrity of the strain’s genome was not fully ensured. In this respect, we believe that the main scientific outcome of our work is that the results presented here provide the most detailed and comprehensive description of the new genetic phenomenon we encountered, the mechanisms of which should be the subject of future studies.

## Figures and Tables

**Table 1:** Primers used for PCR analysis of *MAT* loci in the genome of *K. kurtzmanii* strains.

Primer Name	5′–3′ Sequence	Primer Specificity
A1F	GGAGGTATATGATTGGTGCTAT	Specific for DIC1 terminus in active MAT
T1F	ATCATCCAGCATCCAGATTTTG	Specific for DIC1 terminus in telomere MAT
HR	GGATGTTAATTACCCTGTTATCCCTA	Specific for HIS4
aR	AACTCTGGGATCTTTGGA	Specific for MATa
αR	CGCTTGAGACATGAAAACTG	Specific for MATalpha
TA1R	CAGCAGGAATTACAGACCCTTTCTT	Specific for SLA2 terminus in both MAT
T2F	ATTAGGAGCCTCGAGTTTAAAGG	Specific for DIC1 terminus in telomere MAT in strain 2-1 only

The schemes of primer annealing are presented in Figure 3.

**Table 2:** Description of plasmids used in this study *.

Name	5′–3′ Sequence
pUC19m_(sce)	Contains an integrative fragment *AOX1::(sce-HIS4-sce)* (Supplementary Figure S1A) targeting integration into the distal promoter region of the *K. kurtzmanii P_AOX1_* promoter, without inactivation of *AOX1*.
pPA7272_I-SceI	Autonomously replicating plasmid (Supplementary Figure S1B) provides expression of the meganuclease I-SceI gene under the control of the *FDH* promoter of *K. kurtzmanii* (FDH gene PAS_chr3_0932) [26].
pUC19m_∆MAT	Contains an integrative fragment *mat:::(sce-HIS4-sce)* (Figure 2A) targeting integration into the *MAT* locus of the yeast genome with mating type gene replacement.
pPH7272_βgal	An integrative plasmid (see Supplementary Figure S1) provides the synthesis and secretion of β-galactosidase under the control of the *PAOX1* promoter of *K. kurtzmanii*.

* annotated maps and sequences of plasmids and integration cassettes can be found on the Benchling platform: https://benchling.com/tina_petrova/f_/Be58tktL-construction-of-heterothallic-strains-of-komagataella-kurtzmanii/ (accessed on 5 December 2024).

**Table 3:** The *K. kurtzmanii* strains employed in this study.

Name	Genotype	Reference
Y-727 *his4Δ arg4Δ*	*his4Δ arg4Δ* ^1^	[27]
Y-727(sce)	*his4Δ arg4Δ AOX1::(sce-HIS4-sce)*	this work
Y-727(sce/I-SceI)	*his4Δ arg4Δ AOX1::(sce-HIS4-sce)* [pPA7272_I-SceI]	this work
727-2	*his4Δ arg4Δ mata::(sce-HIS4-sce)*	this work
727-9	*his4Δ arg4Δ matα::(sce-HIS4-sce)*	this work
727-2(I-SceI)	*his4Δ arg4Δ mata::(sce-HIS4-sce)* [pPA7272_I-SceI]	this work
727-9(I-SceI)	*his4Δ arg4Δ matα::(sce-HIS4-sce)* [pPA7272_I-SceI]	this work
Y-727-2(alpha)-1, Y-727-2(alpha)-2, Y-727-2(alpha)-3	*α his4Δ arg4Δ mataΔ*	this work
Y-727-9(a)-1, Y-727-9(a)-2, Y-727-9(a)-3	*a his4Δ arg4Δ matαΔ*	this work
Y-727(His + arg-)	*arg4Δ*	this work
Y-727(Arg + his-)	*his4Δ*	this work
Y-727α(His + arg-)	*α arg4Δ mataΔ*	this work
Y-727α(Arg + his-)	*α his4Δ mataΔ*	this work
Y-727**a**(His + arg-)	*a arg4Δ matαΔ*	this work
Y-727**a**(Arg + his-)	*a his4Δ matαΔ*	this work

^1^ The auxotrophic strain used in this work is a derivative of the original strain VKPM Y-727 described in detail by Naumov et al. [30]. The strain possesses extensive deletions in the coding sequences of the chromosomal genes *his4* and *arg4*. In addition, a fragment of the *S. cerevisiae* Ty1 structural gene, which is used as a target for plasmid DNA integration, was integrated into the region of the *his4* gene.

**Table 4:** Effect of methods of induction of I-SceI meganuclease synthesis on the efficiency of HIS4 gene elimination in Y-727(sce/I-SceI) transformant cells.

Method of Methanol Induction	Methanol Induction	The Proportion of Auxotrophic His- Strains (%) *
Method 1	-	0
	+	75 ± 10
Method 2	-	0
	+	0

* The experiment was performed in two replicates (i.e., two plates). A total of 50–100 colonies were examined for each plate.

**Table 5:** Elimination of the *sce-HIS4-sce* fragment from the genome of strains 727-2 and 727-9 by the action of the I-SceI meganuclease.

Recipient Strain	Transformation by pPA7272_I-SceI	Methanol Induction	Share of Auxotrophic Strains (%) *
*Y*727-2*(alpha)*	-	+	0
	+	-	ND **
	+	+	74
*Y*727-9*(a)*	-	+	0
	+	-	15
	+	+	60

* A total of 50–100 colonies were examined. ** ND—not determined.

**Table 6:** Length of de novo-assembled chromosomes of studied strains, bp.

Chromosome	*K. phafii GS115,* *GCF_000027005*	*K. phafii CBS7435,* *GCA_900235035.2*	Y727 *his4Δ arg4Δ*	Y727-2(alpha)-1	Y727-9(a)-1
Chromosome 1	2,798,491	2,895,357	2,879,939	2,880,076	2,879,832
Chromosome 2	2,394,163	2,396,459	2,609,654	2,606,146	2,609,104
Chromosome 3	2,245,428	2,263,464	2,239,956	4,117,897	2,226,175
Chromosome 4	1,778,296	1,827,946	1,847,874	-	1,822,583

**Table 7:** Analysis of the ability of strains to self-diploidize using diethyl ether.

Strain	No Treatment	Diethyl Ether Treatment
Y727 *his4Δ arg4Δ*	+	+
Y727*-2(alpha)-1*	+	-
Y727*-9(a)-1*	+	-

**Table 8:** Analysis of the ability of strains derived from Y-727 his4Δ arg4Δ, Y-727α and Y-727a to self-diploidize and hybridize.

Arg + His- Derivatives	Y-727 His + arg-	Y-727α His + arg-	Y-727a His + arg-
Y-727 *his4Δ arg4Δ*	+	+	+
Y-727-2(alpha)	+	-	+
Y -727-9(a)	+	+	-

## Data Availability

All genomic data related to the project, including sample description, sequencing reads and genomic assemblies, were deposited to the NCBI database under the BioProject PRJNA1162036. The plasmid maps are available at Benchling platform https://benchling.com/tina_petrova/f_/Be58tktL-construction-of-heterothallic-strains-of-komagataella-kurtzmanii/ (accessed on 5 December2024).

## Supplementary Materials

The following supporting information can be downloaded at: https://www.mdpi.com/article/10.3390/biom15010097/s1. Supplementary text: PCR analysis of chromosomal rearrangements in the obtained strains; Supplementary Table S1. Full stats of de novo assembly results of parental (Y727 *his4Δ arg4Δ*) and heterothallic strains of *K.kurtzmanii*; Figure S1. A. Schematic of the *AOX1::(sce-HIS4-sce)* DNA fragment integrated into the distal promoter region of the *AOX1* gene promoter. B. Map of the episomal plasmid pPA7272_I-SceI; Figure S2. Detailed map of the integrative fragment *AOX1::(sce-HIS4-sce)*; Figure S3. Detailed map of the integrative fragment *mat:::(sce-HIS4-sce)*; Figure S4. Map of plasmid pPH7272_βgal; Figure S5. PCR analysis of the *AOX1* locus of strain Y-727(*sce*) with the integrated *AOX1::(sce-HIS4-sce)* fragment; Figure S6. PCR analysis of the *MAT* loci of the chromosomal DNA of heterothallic strains Y727-2(alpha)-1,2,3, Y727-9(a)-1,2,3 and the parental control strain Y-727 *his4Δ arg4Δ* of *K. kurtzmanii*; Figure S7. Hybridization analysis of the strains Y-727(His+arg-), Y-727(Arg+his-), Y727-2(alpha)-1 (His+arg), Y727-2(alpha)-1 (Arg+his-), Y727-9(a)-1 (His+arg-) and Y727-9(a)-1 (Arg+his-); Figure S8. Original image of the gels shown on A and B panels of the Figure 3 in the main text; Figure S9. Original image of the gels shown on C and D panels of the Figure 3 in the main text; Figure S10. Original image of the pulse-field electrophoresis gel shown on the Figure 4D; Figure S11. Original image of the protein electrophoresis shown on the Figure 5 of the main text.
