# Supplementary material for: The Construction of Heterothallic Strains of Komagataella kurtzmanii Using the I-SceI Meganuclease
Source: Biomolecules. 2025 Jan 10;15(1):97. doi: 10.3390/biom15010097 (PMC11764356; doi:10.3390/biom15010097)
Supplement: Supplementary file 1 [file biomolecules-15-00097-s001.zip › biomolecules-3279196-supplementary.pdf]

SUPPLEMENTARY MATERIALS

The construction of heterothallic strains of Komagataella kurtzmanii using the I-SceI meganuclease

Daria D. Sokolova, Philipp I. Akentyev, Kristina O. Petrova, Aleksei A. Korzhenkov, Irek I. Gubaidullin, Stepan V. Toshchakov * and Dmitriy G. Kozlov *

National Research Center "Kurchatov Institute", 123182 Moscow, Russia;
* Correspondence: toschakov_sv@rrcki.ru or stepan.toshchakov@gmail.com (S.V.T.); kozlov_dg@nrcki.ru or dik-sent@yandex.ru (D.G.K.)

SUPPLEMENTARY TEXT

PCR analysis of chromosomal rearrangements in the obtained strains

A comparative analysis of chromosomal rearrangements was conducted on strains Y727-2(alpha)-1, Y727-2(alpha)-2, Y727-2(alpha)-3 and Y727-9(a)-1, Y727-9(a)-2, Y727-9(a)-3, as well as the original parental strain Y-727 *his4Δ arg4Δ.* The strains were grown on a rich YPD medium. The contents of the *MAT* loci were amplified by PCR and subsequently typed by restriction mapping. Amplicons of the active and telomeric *MAT* loci of the strains were obtained using primer pairs A1F-TA1R and TA1R-T1F/T2F, respectively (Table 1 of the manuscript). Subsequently, the amplicons were subjected to restriction analysis using MluI and EcoRV restriction enzymes (Supplementary Figure 4).

The comparative analysis demonstrated that the characteristics of the amplicons of the active and telomeric *MAT* loci of strains Y727-9(a)-1, Y727-9(a)-2 and Y727-9(a)-3 were fully consistent. The amplicons of the *MAT**a*** allele genes were present in their composition and exhibited no visible differences. Similarly, the amplicons of the active *MAT* locus in strains 2-1, 2-2, and 2-3 displayed no visible differences and exhibited characteristics that corresponded to the presence of *MATα* allele genes in their composition. However, the amplification of the telomeric *MAT* loci sequences of strains Y727-2(alpha)-1, Y727-2(alpha)-2, Y727-2(alpha)-3 using primers T1F and TA1R was unsuccessful. Taking into the account the chromosome fusion detected by *de novo* sequencing of Y727-2(alpha)-1 strain, primer T2F was designed to the subtelomeric region of chromosome 3. Following PCR with primer TA1R annealing to the SLA2 gene and subsequent digestion of the amplicon by EcoRV/MluI endonucleases, the expected fragments were yielded, with lengths of 2025, 1602, 807, and 587 bp, as anticipated (Figure 4F) for all hetherothallic strains, Y727-2(alpha)-1,2,3 (Supplementary Figure 4F).

Thus, the results obtained provided unambiguous confirmation of the desired unification (duplications) of *MAT**a*** genes in the active and telomeric *MAT* loci of strains 9-1, 9-2, and 9-3. This indicated that homologous recombination mechanisms were employed during chromosomal DNA repair in the cells of these strains, rather than nonhomologous end joining (NHEJ). However, while the results of PCR analysis confirmed the identity of the structure of the active *MATα* loci of all three strains (2-1, 2-2, and 2-3), the state of the telomeric *MAT* loci of these strains remained undetermined. To elucidate the nature of the genetic rearrangements, nanopore sequencing and *de novo* assembly of the genomes of strains 2-1, 9-1 and the original strain Y-727 were conducted.

SUPPLEMENTARY TABLES

Supplementary Table S1. Full stats of *de novo* assembly results of parental (Y727 *his4Δ arg4Δ*) and heterothallic strains of *K.kurtzmanii*

genetic element	Y727 *his4Δ arg4Δ*	Y727-2(alpha)-1	Y727-9(a)-1
Chromosome 1	2,879,939	2,880,076	2,879,832
Chromosome 2	2,609,654	2,606,146	2,609,104
Chromosome 3	2,239,956	4,117,897	2,226,175
Chromosome 4	1,847,874	-	1,822,583
amplified mtDNA	94,398	94,521	65,047<br>81,250,<br>39,300
rDNA	120,781	105,545	117,110

SUPPLEMENTARY FIGURES

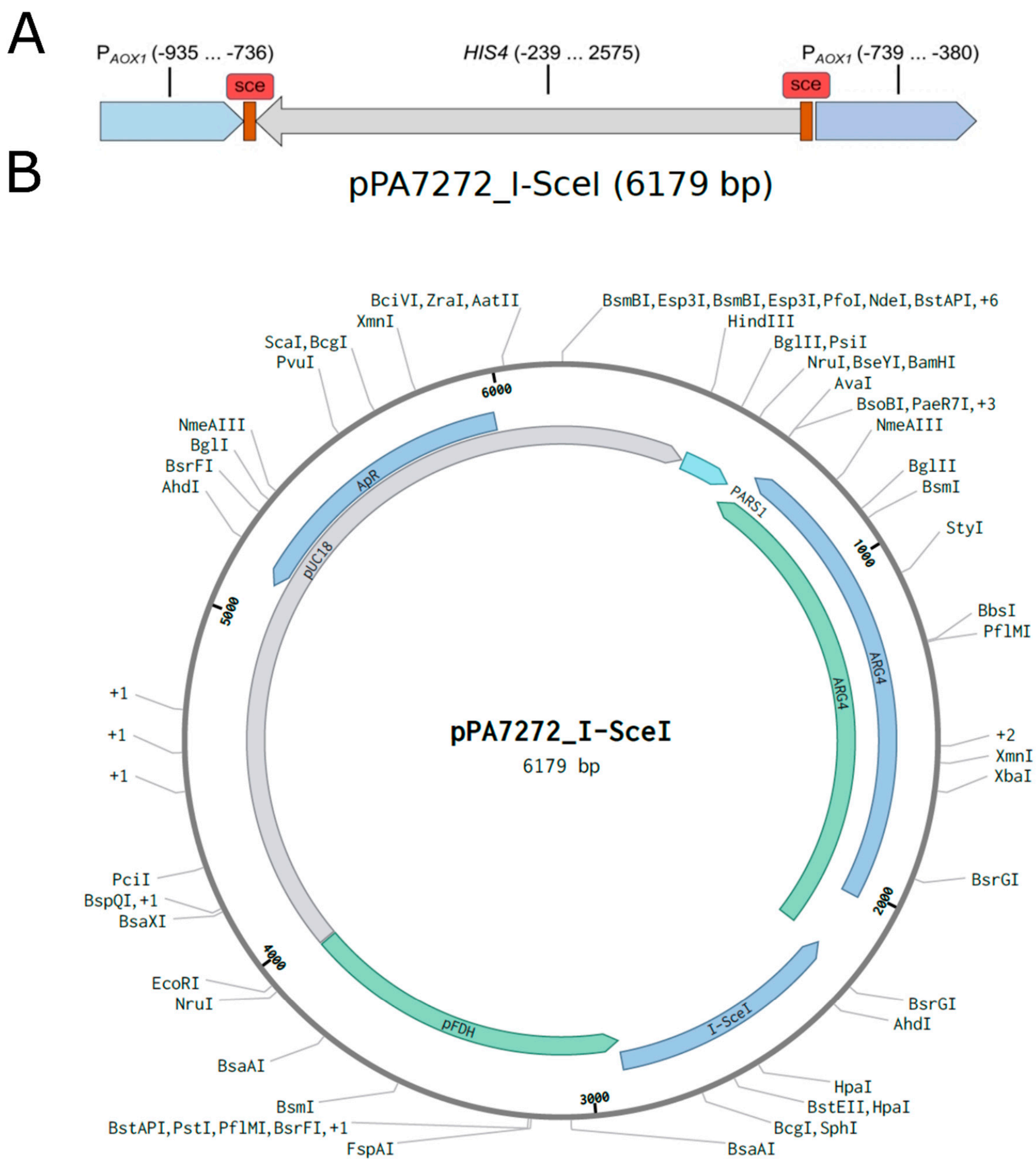

Supplementary Figure S1.

A. Schematic of the *AOX1::(sce-HIS4-sce)* DNA fragment integrated into the distal promoter region of the *AOX1* gene promoter. The fragment contains the *HIS4* gene of *K. kurtzmanii* flanked by I-SceI meganuclease recognition sites *(sce)* and P_{AOX1} promoter sequences designated P_{AOX1}-5‘ and P_{AOX1}-3’. The positions of the 3’-ends of the P_{AOX1}-5‘, *HIS4* and P_{AOX1}-3’ elements relative to the ATG(+1) start codon of the corresponding gene are indicated in parentheses;

B. Map of the episomal plasmid pPA7272_I-SceI, which provided transient expression of the I-SceI meganuclease in *K. kurtzmanii* cells. The plasmid contains the yeast replication initiation region PARS1, a marker *ARG4* gene, and a synthetic I-SceI endonuclease gene under the control of the methanol-

inducible promoter of the formate dehydrogenase *FDH* gene. The plasmid also contains a bacterial part, including the ampicillin resistance gene ApR and ORI replication origin.

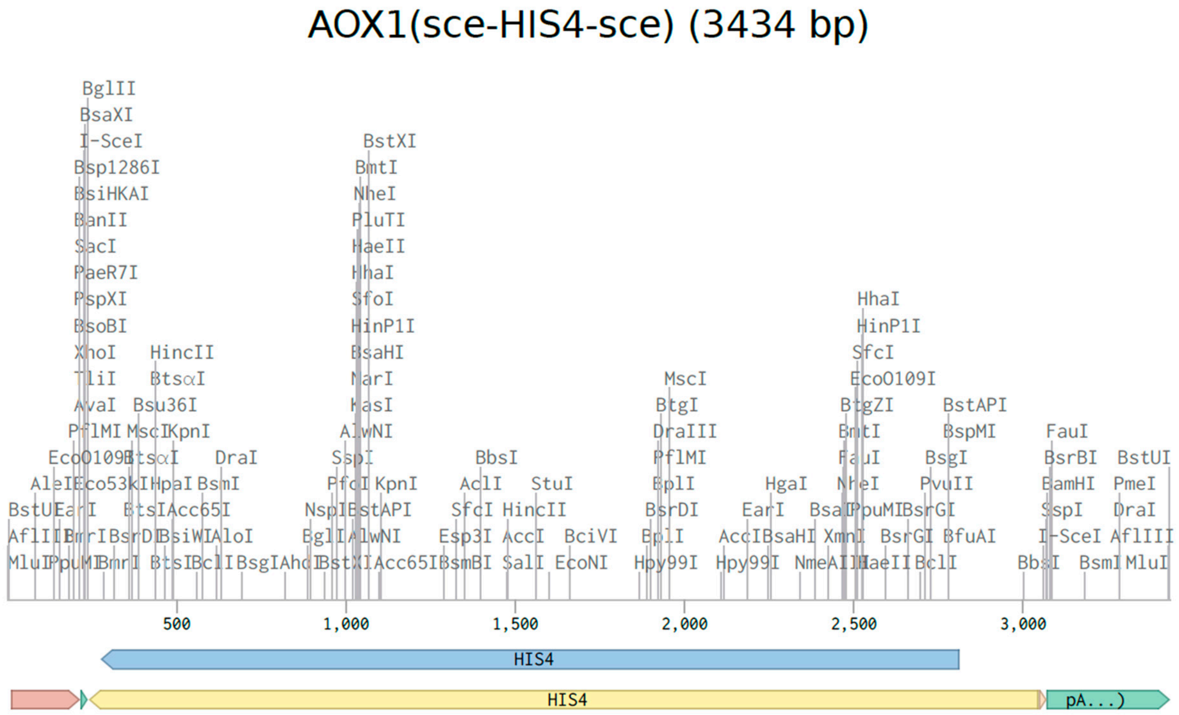

Supplementary Figure S2. Detailed map of the integrative fragment *AOX1::(sce-HIS4-sce)*

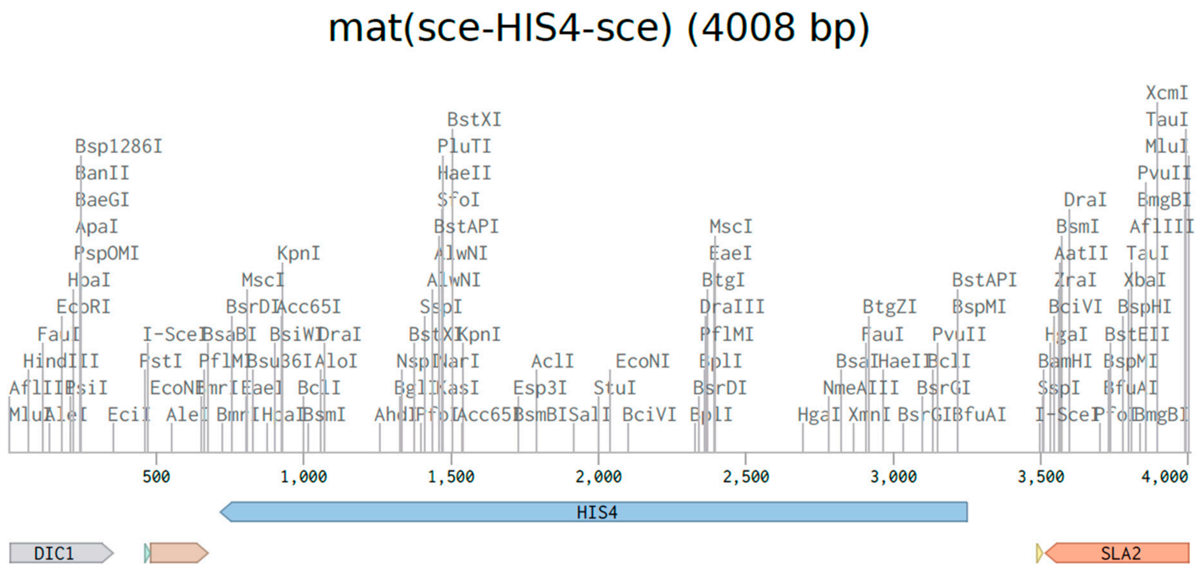

Supplementary Figure S3. Detailed map of the integrative fragment *mat:::(sce-HIS4-sce)*.

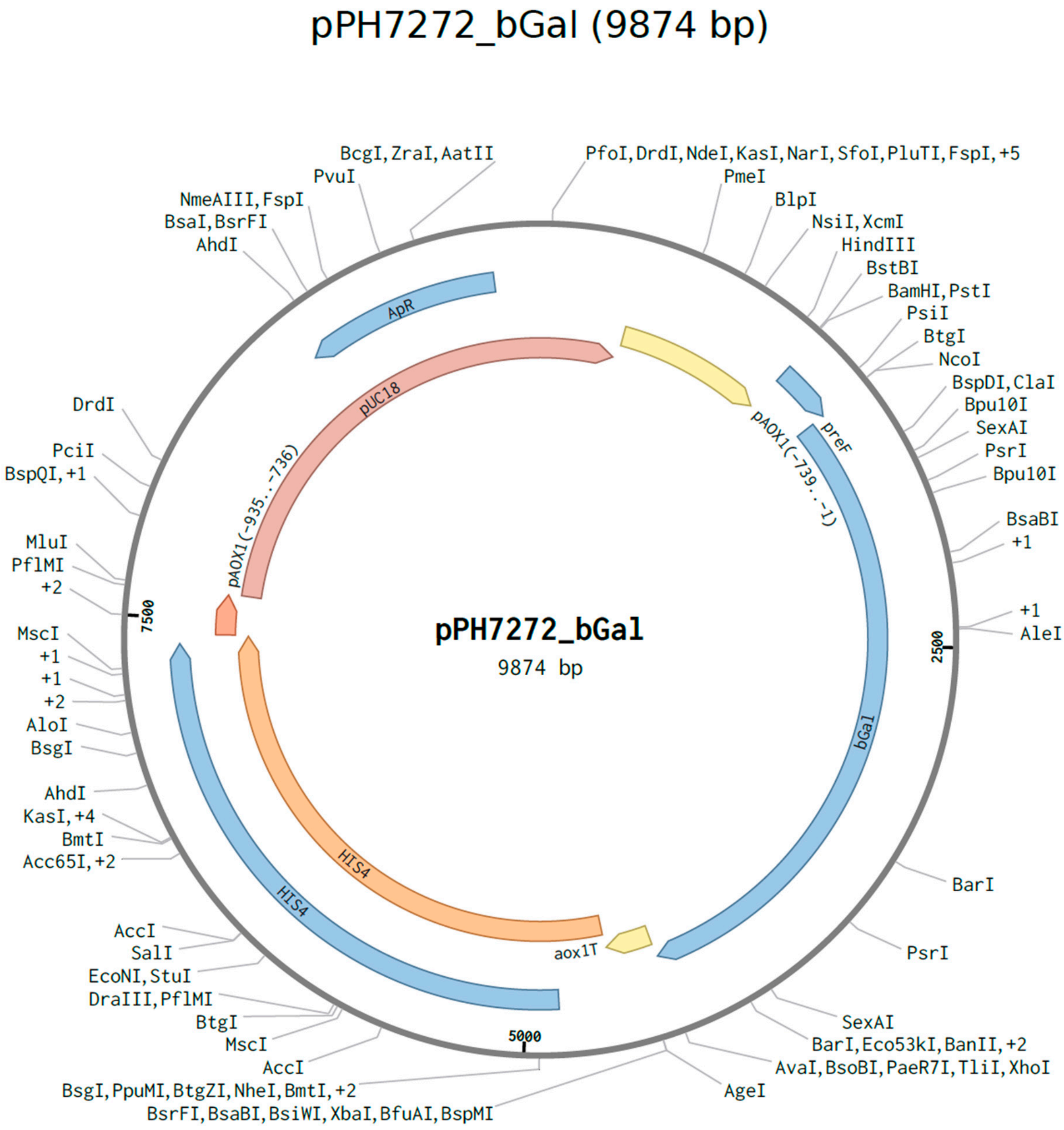

Supplementary Figure S4. Map of plasmid pPH7272_βgal. The integration of the target fragment with the β-galactosidase gene (bGal) is conducted in the distal region of the *AOX1* gene promoter, without compromising its functional integrity, through the use of homology regions designated AOX1(727)proxy and AOX1(727)distal. preF is the standard leader region of yeast S. cerevisiae alpha-factor, enabling β-galactosidase secretion. *HIS4* is a selective marker gene of *K. kurtzmanii*; aox1T, terminator of the *AOX1* gene of *K. kurtzmanii*; AmpR - ampicillin resistance gene; ori - replication origin.

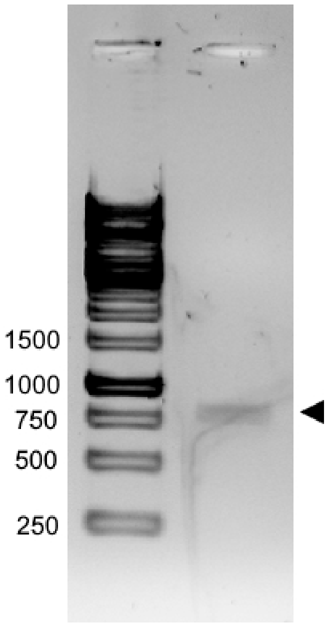

Supplementary Figure S5. PCR analysis of the AOX1 locus of strain Y-727(sce) with the integrated AOX1::(sce-HIS4-sce) fragment (Table 3, Supplementary Figure 1A). The analysis was performed using primer N700 (5'-gaaatatatggtgttgttgggggaaccaaccaagcgagaga) specific for HIS4 and primer N1787 (5'-ctcaagttgttgtgtcgttaaaaaagtcgtgttaaatc) specific for the AOX1 promoter. The calculated size of the DNA fragment to be amplified was 886 nt. The results confirm the targeted integration of the cassette.

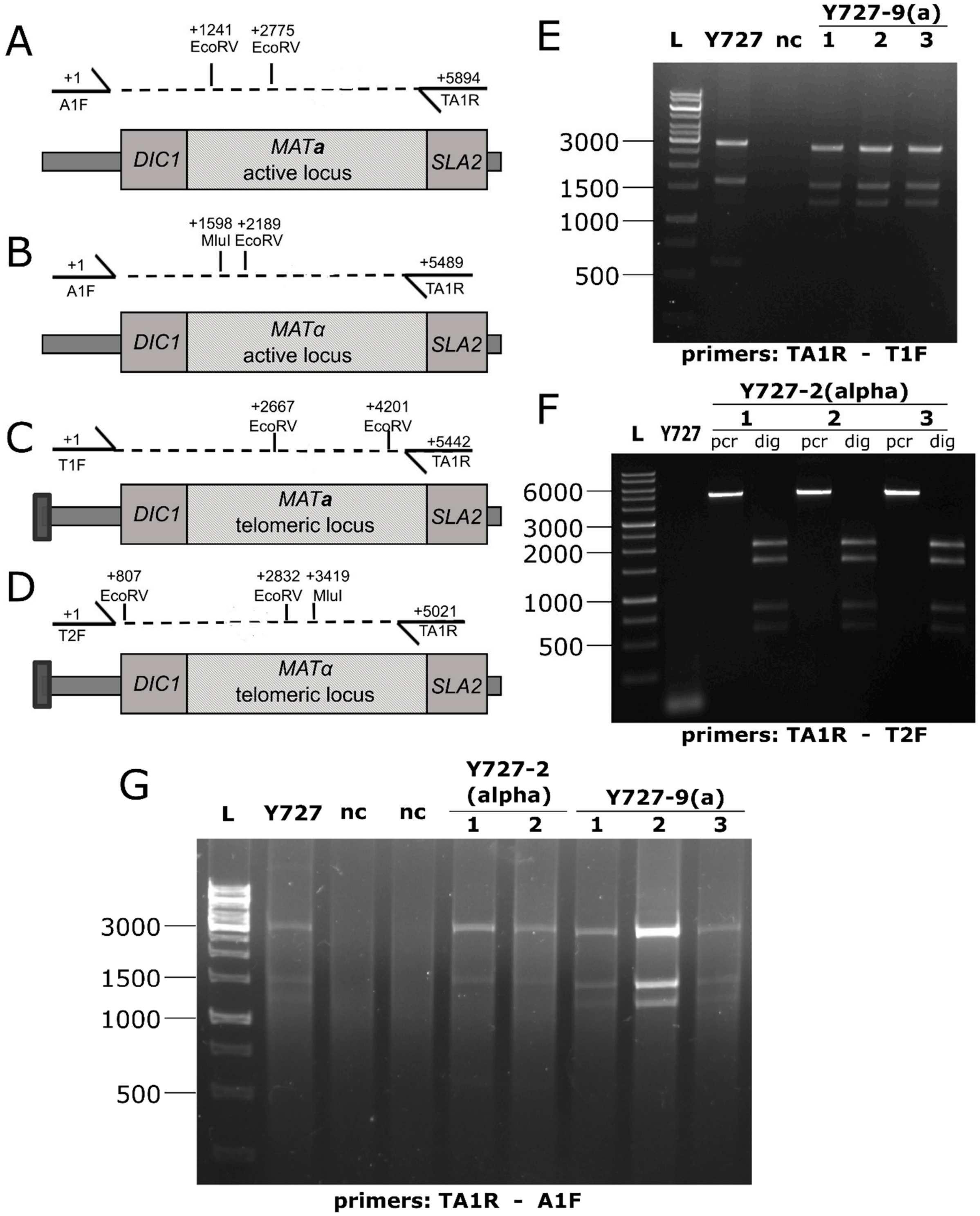

Supplementary Figure S6. PCR analysis of the MAT loci of the chromosomal DNA of heterothallic strains Y727-2(alpha)-1,2,3, Y727-9(a)-1,2,3 and the parental control strain Y-727 his4Δ arg4Δ of *K. kurtzmanii*.

A-D, Structure of the active (A, B) and 'telomeric' (C, D) MAT loci of yeast strains Y727-2(alpha)-1,2,3 (B,D) and Y727-9(a)-1,2,3 (A,C). The positions corresponding to the 5' end residues of the primers and position of restriction sites are indicated.

E, Gel image of the PCR analysis of telomeric MAT locus of strains Y727-9(a)-1,2,3. Restriction of amplified fragments was performed with EcoRV-MluI. Expected band sizes: Y-727 - 2847, 1598, 591 bp; Y727-9(a)-1,2,3 - 2667, 1534, 1241 bp. L - GeneRuler 1 kb DNA Ladder (Thermo Fisher Scientific, Waltham, MA, USA)

F, Gel image of the PCR analysis of telomeric MAT locus of strains Y727-2(alpha)-1,2,3. Restriction of amplified fragments was performed with EcoRV-MluI. Expected band sizes: 2025, 1602, 807 and 587 bp. Expected size of undigested amplicon - 5021 bp. L - DNA Ladder 1 kb (Evrogen, Russia).

G, Gel image of the PCR analysis of active MAT locus of strains Y727-2(alpha)-1,2, Y727-9(a)-1,2,3 and the parental control strain Y-727 his4Δ arg4Δ. Restriction of amplified fragments was performed with EcoRV-MluI. Expected band sizes: Y727-2(alpha)-1,2 - 3300, 1598 and 591 bp; Y727-9(a)-1,2,3 - 3119, 1534, 1241. L - GeneRuler 1 kb DNA Ladder (Thermo Fisher Scientific, Waltham, MA, USA)

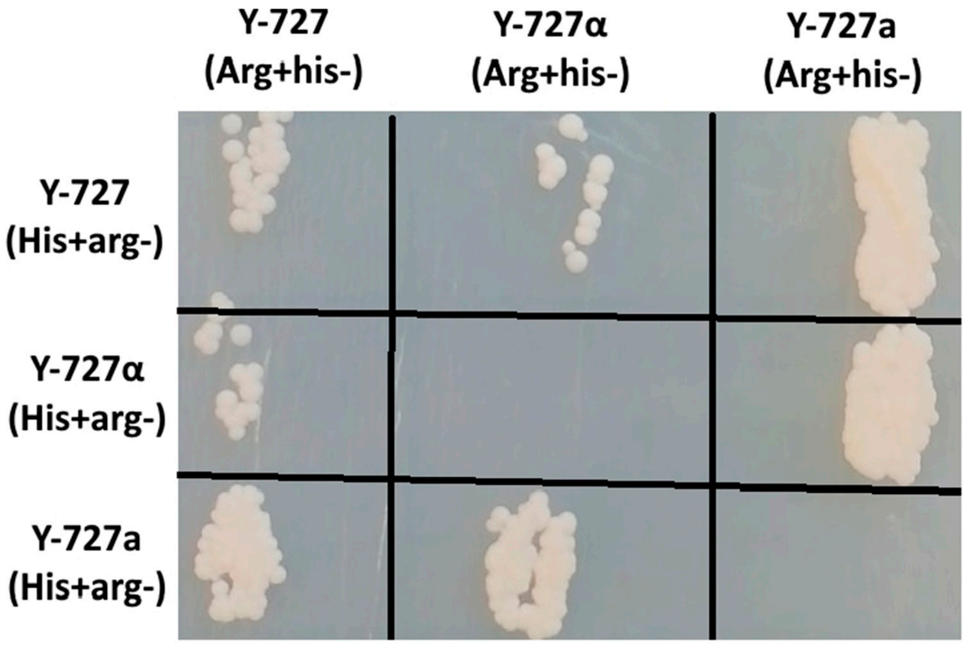

Supplementary Figure S7. Hybridization analysis of the strains Y-727(His+arg-), Y-727(Arg+his-), Y727-2(alpha)-1 (His+arg), Y727-2(alpha)-1 (Arg+his-), Y727-9(a)-1 (His+arg-) and Y727-9(a)-1 (Arg+his-).

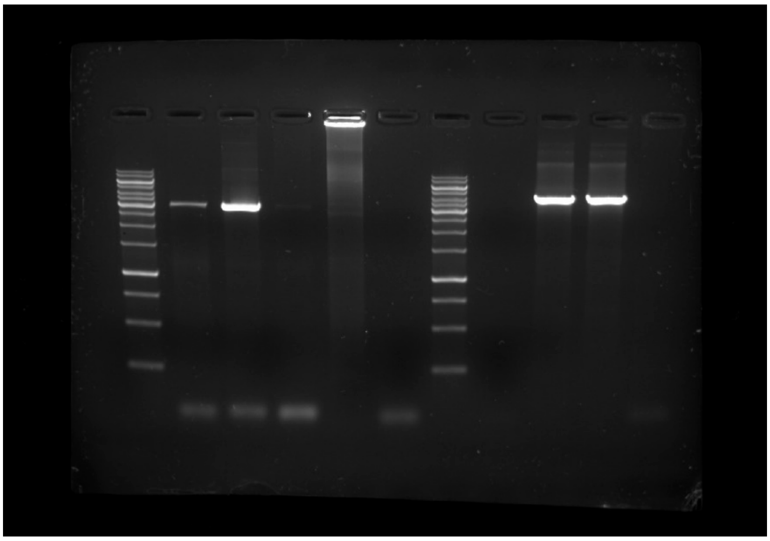

Supplementary Figure S8. Original image of the gels shown on A and B panels of the Figure3 in the main text.

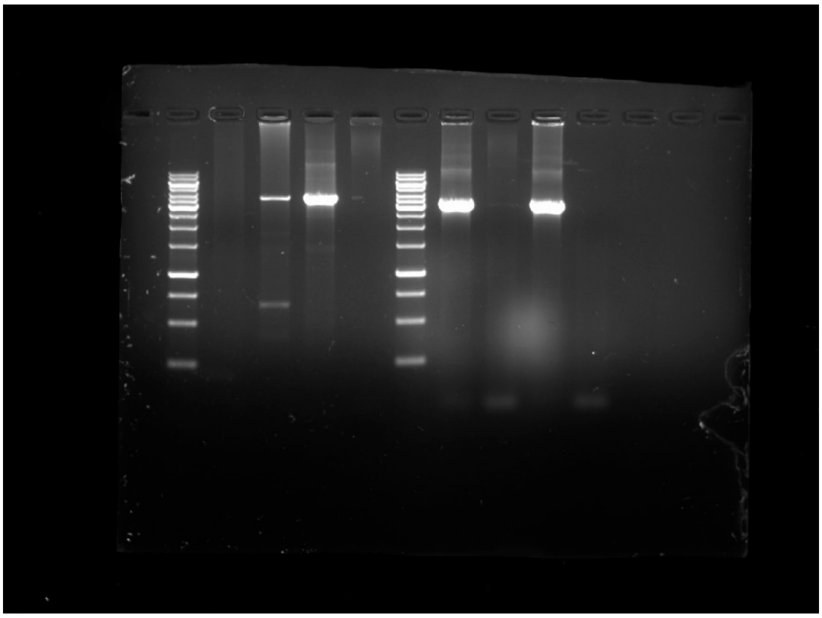

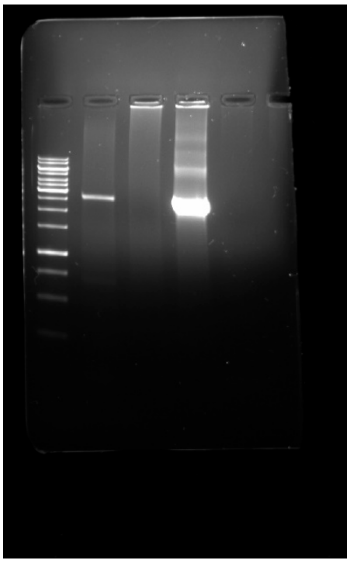

Supplementary Figure S9. Original image of the gels shown on C, D and E panels of the Figure3 in the main text.

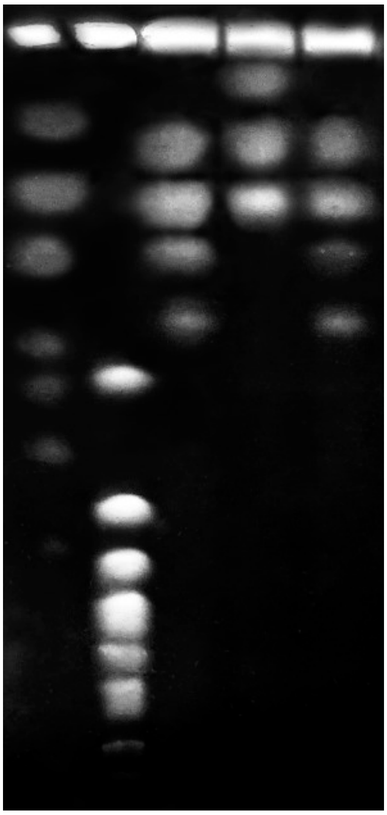

Supplementary Figure S10. Original image of the pulse-field electrophoresis gel shown on the Figure 4D.

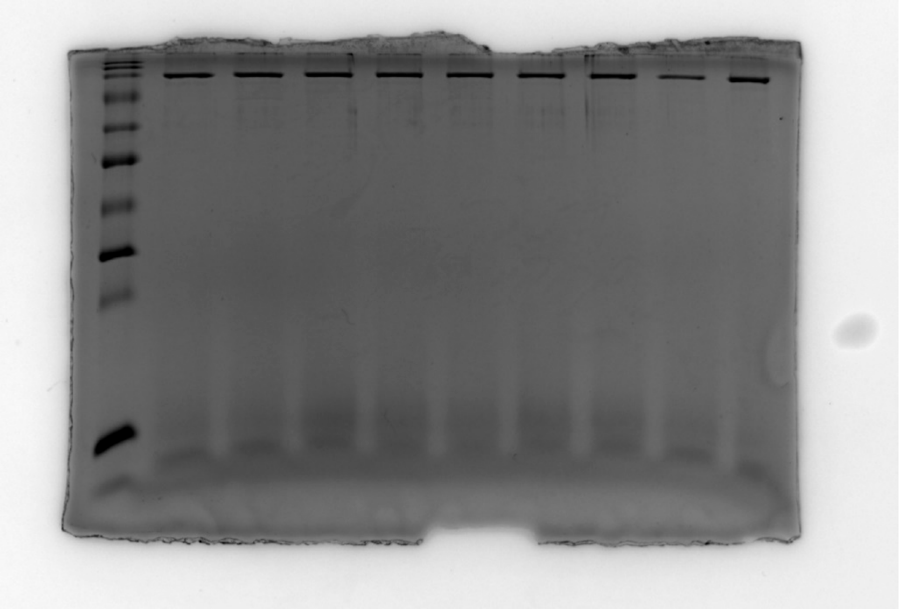

Supplementary Figure S11. Original image of the protein electrophoresis shown on the Figure 5 of the main text.
